# Chinese adolescents’ power distance value and prosocial behavior toward powerful people: A longitudinal study

**DOI:** 10.1371/journal.pone.0208473

**Published:** 2018-12-06

**Authors:** Xinyuan Fu, Yichen Lv, Zhixu Yang, Xiaoxia Yu, Rongrong Wang

**Affiliations:** 1 Department of Psychology, School of Sociology and Psychology, Central University of Finance and Economics, Beijing, China; 2 School of Economics, Central University of Finance and Economics, Beijing, China; 3 Mental Health Education Center, Students’ Affairs Department, North China Electric Power University, Beijing, China; Middlesex University, UNITED KINGDOM

## Abstract

We were interested in how specific cultural value and adolescent social behavior would influence each other over time. Thus the present study explored the longitudinal and bidirectional relations between adolescents’ power distance value and prosocial behavior toward powerful people over a year. A sample of 434 Chinese adolescents participated in the investigation (initial mean age = 11.27; 54.15% females). The results based on cross-lagged models showed that, earlier prosocial behavior toward powerful people was positively correlated to subsequent power distance value, but not vice versa. The findings point toward an understanding of the important role of adolescent social behavior on his/her cultural value development, and also shed light on future research in terms of the interplay between cultural values and individual’s social behaviors in other cultures.

## Introduction

Prosocial development depends on the cultural system in which one grows up [1], and support for this argument comes from a series of cross-cultural studies. For example, residents in India are on average less cooperative than US residents in one-shot interactions in the laboratory setting [2–3]. Previous research has also shown that Indians are more spiteful than Americans when making decisions about real monetary allocations between themselves and another anonymous participant [4]. This empirical evidence suggests that culture plays an important role in prosocial behavior, yet few studies have examined the influences of cultural values on prosocial behavior/development within one specific culture. Thus we tried to explore the effects of cultural values on prosocial behavior based on a sample of Chinese adolescents.

As traditional Chinese culture emphasizes hierarchy, and people are socialized to comply with the obligations and rules attached to their roles in the hierarchical system [5], values like power distance, authority, humility, and wealth are highly important in Chinese culture. In addition, establishing and maintaining interpersonal harmony via prosocial interactions is highly endorsed by Chinese culture (e.g., [6–7]), and this cultural norm is particularly in the case when people tend to establish positive contacts with powerful people for meeting individual needs (e.g., [8]). Therefore, we aimed to examine the effect of power distance value on adolescents’ prosocial behavior toward powerful people. On the other hand, with the enormous economic progress and increasing globalization it has made over the past forty years, Chinese society has been changing dramatically. The economic and social changes correspond to the introduction of individualistic values from the western cultures [9]. These values have been gradually accepted by many Chinese people, especially in the younger generation [1, 10]. In this regard, the subject of the present study would shed some light on understanding adolescents’ social functioning and adjustment in the changing society.

Moreover, little research has been conducted to examine how adolescents’ cultural values might be shaped/influenced by social behaviors, such as prosocial behavior. The culturally guided social interaction processes including prosocial behavior toward powerful people likely serve as an important socialization agent of children’s cultural value development [11]. Thus we tried to extend the existing literature by examining the longitudinal and bidirectional relations between adolescents’ power distance value and prosocial behavior toward powerful people in the present study.

### The role of power distance value on prosocial behavior among Chinese adolescents

Power distance has been defined as the extent to which the less powerful members of organizations and institutions (like the family) accept and expect that power is distributed unequally [12]. Individuals with higher power distance value are more likely to view inequality as a result of different social positions as natural and even desirable than those with lower power distance value [13]. In line with this definition, individuals with higher power distance value usually have more positive attitudes toward the powerful and also higher willingness to interact with the powerful than people with lower power distance value (e.g., [14]). Thus, it may be logical to assume that adolescents with higher power distance value would be more prosocial toward powerful people. In the present study, powerful people refer in particular to parents, teachers, and others who have capacity to influence or control adolescents by providing valued resources [15]. Psychological processes and behaviors are attuned to the particular cultural meaning system with which the individual develops [16]. Growing up in a high power-distance culture, Chinese people are more culturally guided to and then are more inclined to build and maintain positive relationships with powerful people for desired resources or outcomes (e.g., [17]). Because responding prosocially to powerful people is an efficient way to build or maintain positive relationships with them (e.g., [18]), we supposed that, within the Chinese culture adolescents who hold higher power distance value might be more likely to help powerful people than those holding lower power distance value.

### The role of prosocial behavior on Chinese adolescents’ power distance value

Humans interact with and seize meanings from their cultures, and then internalize specific cultural values by these processes [16]. From a very young age, resulting from different experiences, children within one culture might behave differently, think in different ways, and gradually acquire some cultural values to different extents. In line with this reasoning, frequent prosocial interactions with powerful people might help adolescents obtain more desired resources (such as more pocket money from parents), and have more positive relationships with the powerful (e.g., teachers). In this way, adolescents might be more likely to view power differentials as acceptable and socially normative, and consequently hold higher power distance value.

Furthermore, self-perception theory [19] provides a theoretical guidance for us to understand how prosocial behavior toward powerful people might impact adolescents’ power distance value. The theory supposes that people observe their own actions to make inferences about inner states, such as attitudes and values, and it is particularly likely to occur when the behavior is perceived as voluntary. For instance, empirical studies have shown that adolescents’ prosocial practices in the family context could predict increases in familism values [20], and helping strangers is positively correlated to adolescents’ subsequent moral values [21]. On the other hand, intervention research has demonstrated that encouraging pursuing goals that stand in opposition to materialism could effectively inhibit materialistic values among adolescents [22]. All these findings might point toward an assumption that adolescents’ behavior could influence their value development. According to the self-perception theory and the instances, we assumed that adolescents who are more willing to help powerful people would take power distance more seriously in order to rationalize their behavior. With more and more prosocial behaviors toward the powerful, the adolescents might view the inequality among persons in different positions as a natural aspect of the social order, and hold positive beliefs about the power distances between people. Taken together, we expected to see a positive relationship between adolescents’ prosocial behavior toward powerful people and subsequent power distance value.

### The current study

In the present study we were interested in exploring the longitudinal and bidirectional relationships of adolescents’ power distance value and prosocial behavior toward powerful people based on a Chinese sample. This attempt is meaningful in terms of understanding the nature and significance of the interplay between cultural values and adolescents’ social behaviors. We hypothesized that power distance value would be positively and longitudinally associated with adolescents’ prosocial behavior toward powerful people, and in return, earlier prosocial behavior toward powerful people would elicit higher power distance value among Chinese adolescents.

## Methods

### Ethics statement

This study was approved by Institutional Review Board of School of Sociology and Psychology at Central University of Finance and Economics. Written informed consent was obtained from all the parents of the participants.

### Participants

A sample of 543 adolescents ranging from 11 to 14 years old in Beijing, China participated in the investigation at Time 1 and 434 of them participated at Time 2 (235 girls, *M*_age_ = 12.26 years, *SD*_age_ = 0.54), with one year apart. The participants were in the 6^th^ grade (which is the last year of primary school in China) at Time 1 and were in the 7^th^ grade (which is the first year of middle school) at Time 2. It partly explained the relatively low response rate at Time 2, because some non-resident students had to transfer back to the middle schools in the place of their own residence and failed to continue to participate in our research. As described in detail previously [23], 21.43% made less than ¥5,000 regarding monthly family income, 57.14% made between ¥5001 and ¥10,000, 17.74% made between ¥10,001 and ¥20,000, 2.76% made more than ¥20,000, and the remaining 0.92% was missing. The overall proportion of the missing data was less than 5%, and the data set was then treated as missing completely at random (MCAR). Mplus’s maximum likelihood estimation of models under MCAR was used to deal with these missing values.

### Measures

#### Power distance value

Adolescents’ power distance value was assessed using the 4-item power distance subscale of the cultural value orientation scale [24]. The example items included “*I believe older people in the family should make the important decisions and younger ones should not question their decisions*” and “*I believe students should obey their teachers*, *not question them*”. Participants rated the items on a five-point response format from 1 (*strongly disagree*) to 5 (*strongly agree*). The reliabilities at Time 1 and Time 2 were .69 and .75. Latent variables for power distance value at two time points were created. Measurement weak factorial invariance across time was tested [25], and invariance was reached (Δ*χ*^2^ (3) = 6.78, *p* > .05). Factor loadings ranged from .38 to .80. The only item with a factor loading below .40 was “*I believe superiors (e*.*g*., *managers*, *parents*, *teachers) should be able to make important decisions without having to ask for the opinions of others (e*.*g*., *subordinates*, *kids*, *and students*, *respectively)*”. This relatively low factor loading might be due to that adolescents were not so familiar with the “manager-subordinate” dyads, and then failed to give consistent answers.

#### Prosocial behavior toward powerful people

This construct was measured by a modified version of the kindness and generosity subscale of the Values in Action Inventory of Strengths [26]. The present study adapted six of the items to assess prosocial behavior toward powerful people (e.g., “*I enjoy doing favors for powerful people*”). Respondents answered on a 5-point Likert-type scale from 1 (*not like me at all*) to 5 (*very much like me*) in terms of how much they disagreed or agreed with statements about themselves. Latent variables for prosocial behavior toward powerful people at two time points were created. Measurement weak factorial invariance across time was reached (Δ*χ*^2^ (5) = 3.84, *p* > .05). The reliabilities at Time 1 and Time 2 were .91 and .92 with factor loadings ranging from .59 to .94.

#### Demographic information

Adolescents’ gender, age, whether being the only-child in the family or not, parents’ educational levels, and monthly family income were investigated. As for parents’ educational levels, the participants answered on a 5-point scale (1 = primary school, 2 = middle school, 3 = high school, 4 = undergraduate, and 5 = postgraduate). Following the approach of a previous study [27], the present research measured monthly family income via a 5-point scale (1 = below ¥5,000, 2 = ¥5001–10000, 3 = ¥10001–15000, 4 = ¥15001–20000, and 5 = above ¥20000).

### Attrition analyses

Attrition analyses were conducted via a series of *t*-tests and chi-square tests. As described previously [23], boys (*p* < .001) or those with less educated fathers and mothers (*p* < .01; *p* < .001) were more likely to drop out of the investigation at Time 2. Regarding the study variables, adolescents with less prosocial behavior toward powerful people were more likely to drop out (*p* < .05). As the participants were in the 6^th^ grade (which is the last year of primary school in China) at Time 1 and were in the 7^th^ grade (which is the first year of middle school) at Time 2, some non-resident students had to transfer back to the middle schools in the place of their own residence, which resulted in the attrition. These non-resident students who were absent at Time 2 usually had less educated parents and lower social status, which might influence their socialization, and then had less prosocial behavior toward powerful people. Whereas no significant differences (*p*s > .24) on power distance value, and other demographics (age and monthly family income) were found. In sum, though the final sample was more prosocial toward powerful people, more from highly-educated families, and became more female, it did not experience significant attrition in terms of power distance value, participant’s age, and monthly family income.

## Results

### Descriptive statistics and correlations

Descriptive statistics and correlations of the study variables are shown in Table 1. The results indicated that adolescents’ power distance value and prosocial behavior toward powerful people at Time 1 and Time 2 were positively correlated with one another.

**Table 1:** Descriptive statistics and correlations between Chinese adolescents’ power distance value and prosocial behavior toward powerful people.

	01	02	03	04
01. PB_Powerful_1				
02. PB_ Powerful_2	.26**			
03. Power Distance value_1	.21**	.10*		
04. Power Distance value_2	.19**	.11*	.50**	
*M*	3.14	2.88	2.81	2.42
*SD*	1.07	0.99	0.87	0.88

*Note*. PB_Powerful = prosocial behavior toward powerful people, 1 = Time 1, 2 = Time 2.

* *p* < .05,

** *p* < .01.

We then ran repeated measures ANOVAs to test the mean differences over time and the interactions of time point and gender on the study variables. The main effects of both time point and gender on power distance value were significant (time point: *F*(1, 425) = 79.86, *p* < .001, partial eta^2^ = .16, 1-*β* = 1.00; gender: *F*(1, 425) = 7.53, *p* = .006, partial eta^2^ = .17, 1-*β* = .78). Adolescents hold lower power distance value at Time 2 (*M* = 2.42, *SD* = 0.88) than at Time 1 (*M* = 2.81, *SD* = 0.87), and boys had higher power distance value than girls (Time 1: *M*_boys_ = 2.91, *SD*_boys_ = 0.84, *M*_girls_ = 2.72, *SD*_girls_ = 0.89; Time 2: *M*_boys_ = 2.53, *SD*_boys_ = 0.94, *M*_girls_ = 2.33, *SD*_girls_ = 0.81). No interaction effect between time point and gender was found for power distance value (*F*(1, 425) = 0.04, *p* = .84). For prosocial behavior toward powerful people, only the main effect of time point was significant (time point: *F*(1,416) = 15.58, *p* < .001, partial eta^2^ = .04, 1-*β* = .98; gender: *F*(1, 416) = 0.80, *p* = .37; interaction effect: *F*(1, 416) = 0.16, *p* = .69). The adolescents were less likely to help powerful people at Time 2 (*M* = 2.88, *SD* = 0.99) than at Time 1 (*M* = 3.14, *SD* = 1.07).

### Cross-lagged models

Cross-lagged models were implemented using Mplus software with the two waves of adolescents’ power distance value and prosocial behavior toward powerful people. Given the gender differences found above, multiple-group comparisons across adolescent gender were conducted. The Δ*χ*^2^ test suggested that constraining the cross-lagged paths to be identical across gender groups did not elicit a significant decrease in model fit (Δ*χ*^2^ (2) = 1.81, *p* = .40), thus a single-group model was run as the final model. Because of the sample attrition and the differences on the demographics between the retained adolescents and those who were absent at Time 2, gender, parents’ educational levels, as well as monthly family income and whether being the only-child in the family or not were treated as the control variables. The final model had good model fit (*χ*^2^ (256) = 384.49, *p* < .01, CFI = .97, TLI = .97, RMSEA = .04, SRMR = .05).

**Fig 1:**
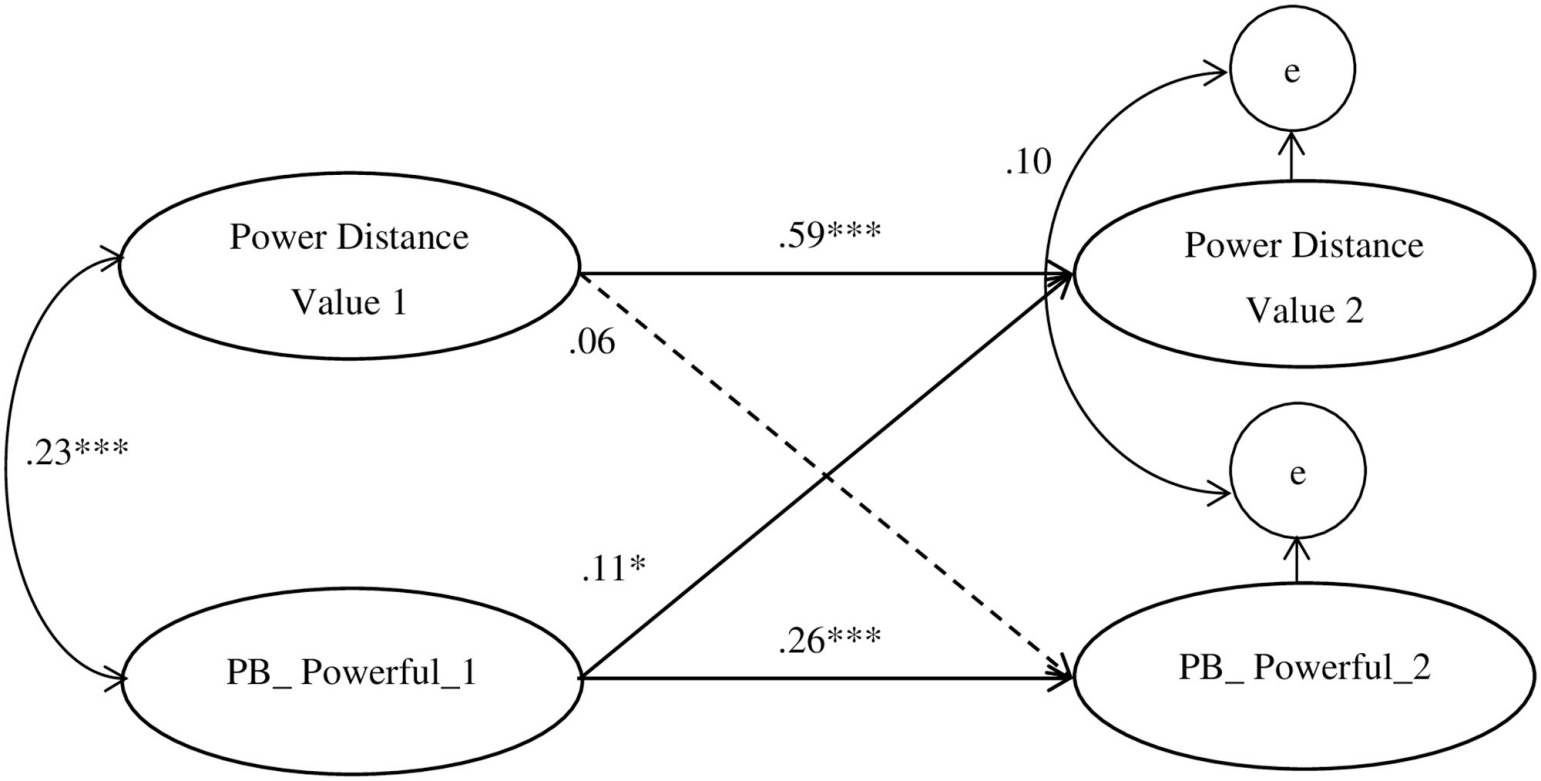
Cross-lagged effects of Chinese adolescents’ power distance value and prosocial behavior toward powerful people. *Note*. PB_Powerful = prosocial behavior toward powerful people, 1 = Time 1, 2 = Time 2. *χ*^2^ (256) = 384.49, *p* < .01, CFI = .97, TLI = .97, RMSEA = .04, SRMR = .05. All paths represent significant standardized beta weights. The dashed lines indicate nonsignificant paths. Gender, parents’ educational levels, monthly family income, and whether being the only-child in the family or not were controlled in the model, but were omitted for parsimony. * *p* < .05, *** *p* < .001.

As shown in Fig 1, adolescents’ power distance value at Time 1 was not significantly associated with prosocial behavior toward powerful people at Time 2 (*β* = .06, *p* = .33). Whereas prosocial behavior toward powerful people at Time 1 was positively associated with power distance value at Time 2 (*β* = .11, *p* = .04). Both the stability paths of power distance value (*β* = .59, *p* < .001) and prosocial behavior toward powerful people (*β* = .26, *p* < .001) from Time 1 to Time 2 were significant. None of the associations between the control variables at Time 1 and the study variables at Time 2 was significant (all *p*s > .05).

## Discussion

The purpose of the current study was to explore how specific cultural value and adolescent social behavior would influence each other over time. By examining the longitudinal and bidirectional relationships between power distance value and prosocial behavior toward powerful people among Chinese adolescents, we found that earlier prosocial behavior toward powerful people was positively correlated to subsequent power distance value, but not vice versa. The findings point toward an understanding of the important role of adolescent social behavior on his/her cultural value development.

### Relation between earlier power distance value and subsequent prosocial behavior

The current research did not find the expected positive effect of power distance value on adolescents’ prosocial behavior toward powerful people. Perhaps it was due to the relatively less salient role of power distance value on predicting prosocial behavior. According to Ajzen’s (1991) Theory of Planned Behavior (TPB) [28], the best predictor of a behavior is intention, which is determined by three factors: the person’s attitudes/values toward the behavior, perceptions of important others’ approval of the behavior (subjective norms), and perceptions of the ease or difficulty of performing the behavior (perceived behavioral control). Although values motivate behavioral choices and actions [29], power distance value might be not one of the dominated factors in predicting prosocial behavior, as prosocial behavior seems to be highly associated with adolescents’ dispositional traits (e.g., sympathy, self-regulation, and self-esteem) and other-oriented values like emphasizing promoting others’ welfare (e.g., [21, 30, 31]).

Moreover, with the rapid economic and social changes in China, Chinese traditional values have been merging with the western individualistic values and ideologies such as independence, which is especially the case for children and adolescents (e.g., [27]). This changing nature of Chinese cultural values (particularly among the younger generation) might also partly explain the nonsignificant correlation between earlier power distance value and subsequent prosocial behavior toward powerful people. On the other hand, as adolescents’ values are somehow malleable and are still developing, power distance value during early adolescence (the current sample consisted of 11- to 14-year-olds) might have an emerging effect on prosocial behavior, but the relatively short-term one-year design of the present study did not allow us to see significant changes induced by power distance value. Further research is needed to explore more waves of data to examine these patterns into middle and late adolescence.

### The effect of prosocial behavior toward powerful people on power distance value

Consistent with our assumption, prosocial behavior toward powerful people at Time 1 was positively correlated to power distance value at Time 2. Based on the self-perception theory and given the formation nature of values during adolescence, adolescents are inclined to internalize and develop their values by observing and interpreting their behaviors. Because Chinese adolescents are socialized to treat superiors (e.g., parents, teachers, peer leaders) seriously and rightly [32], conducting prosocial behavior toward powerful people can serve as an important external cue for them to reason their attitudes and values about power differentials.

By helping powerful people, adolescents might gain potential profits from the powerful ones. For instance, those students always doing some favors for teachers would be more likely to be liked and trusted by their teachers [33–34], which is very common in Chinese schools. Gradually, those adolescents having frequent prosocial interactions with the powerful would believe that treating powerful people prosocially would bring benefits and superiorities in many aspects [35]. Finally, adolescents who preferred to help powerful people would get used to the differences between people with different power levels, and be more willing to obey the cultural norms emphasizing power differentials. In this way, prosocial behavior toward powerful people facilitated the socialization of adolescents’ power distance value.

### Gender difference regarding prosocial behavior toward powerful people

No significant gender difference on prosocial behavior toward powerful people was found in our research, though most of the former studies have shown that females are significantly more prosocial than males. For example, several meta-analyses indicated that females are more altruistic in Dictator Game experiments [36–37], are more cooperative in social dilemmas [38], and are more honest in Sender-Receiver games [39] than males. However, there is also empirical evidence showing that adolescent boys and girls have the same levels of prosocial behavior toward family, whereas girls are more prosocial toward friends and strangers than boys [23]. One explanation of the mixed findings is that the role of gender on prosociality might vary across the target toward whom prosocial behavior is directed. In this regard, future research is desirable to test how gender differences in prosociality would vary as a function of target.

### Limitations and contributions

Limitations of this study are addressed. First, attrition analyses showed that the retained adolescents were more prosocial toward powerful people than those who were absent at Time 2, which restricted to some extent the generalization of the findings. Second, as the current study was based on a sample of Chinese adolescents who were growing up in a relatively high power distance culture, such studies need to be carried out in other cultures with different levels of power distance to generalize the findings. Even so, the present research has important contributions by figuring out the longitudinal relations between power distance value and prosocial behavior toward powerful people among adolescents. It provides clear evidence for the significant role of prosocial behavior toward powerful people on the development of adolescent power distance value. More broadly, the findings would enlighten future research in terms of understanding the nature and significance of the interplay between cultural values and individual’s social behaviors.

## Supporting information

S1 File The raw data of this study. (SAV)

## References

[1] Chen X, Cen G, Li D, He Y. Social functioning and adjustment in Chinese children: The imprint of historical time. Child Dev. 2005;76(1):182–95. 10.1111/j.1467-8624.2005.00838.x 15693766 10.1111/j.1467-8624.2005.00838.x

[2] Capraro V, Cococcioni G. Social setting, intuition and experience in laboratory experiments interact to shape cooperative decision-making. Proc Biol Sci. 2005;282(1811):1003–7. 10.1098/rspb.2015.0237 26156762 10.1098/rspb.2015.0237 PMC4528537

[3] Capraro V, Jordan JJ, Rand DG. Heuristics guide the implementation of social preferences in one-shot Prisoner’s Dilemma experiments. Sci Rep. 2014;4:6790 10.1038/srep06790 25348470 10.1038/srep06790 PMC4210943

[4] Capraro V, Corgnet B, Espín AM, Hernán-González R. Deliberation favours social efficiency by making people disregard their relative shares: Evidence from USA and India. R Soc Open Sci. 2017;4(2):160605 10.1098/rsos.160605 28386421 10.1098/rsos.160605 PMC5367314

[5] Schwartz S H. A theory of cultural value orientations: Explication and applications. Comp Sociol. 2006;5(2):137–82.

[6] Wei M, Su JC, Carrera S, Lin SP, Yi F. Suppression and interpersonal harmony: A cross-cultural comparison between Chinese and European Americans. J Couns Psychol. 2013;60(4):625–33. 10.1037/a0033413 23978268 10.1037/a0033413

[7] Zhang YB, Lin MC, Nonaka A, Beom K. Harmony, hierarchy and conservatism: A cross-cultural comparison of Confucian values in China, Korea, Japan, and Taiwan. Commun Res Rep. 2005;22(2):107–15. 10.1080/00036810500130539

[8] Dunning JH, Kim C. The cultural roots of guanxi: An exploratory study. World Econ. 2007;30(2):329–41. 10.1111/j.1467-9701.2007.00885.x

[9] Chen X, French DC. Children’s social competence in cultural context. Annu Rev Psychol. 2008;59:591–616. 10.1146/annurev.psych.59.103006.093606 18154504 10.1146/annurev.psych.59.103006.093606

[10] Tamis-LeMonda CS, Way N, Hughes D, Yoshikawa H, Kalman RK, Niwa EY. Parents’ goals for children: The dynamic coexistence of individualism and collectivism in cultures and individuals. Soc Dev. 2008;17(1):183–209. 10.1111/j.1467-9507.2007.00419.x

[11] Tyler KM, Dillihunt ML, Boykin AW, Coleman ST, Scott DM, Tyler CMB. et al Examining cultural socialization within African American and European American households. Cultur Divers Ethnic Minor Psychol. 2008;14(3):201–4. 10.1037/1099-9809.14.3.201 18624584 10.1037/1099-9809.14.3.201

[12] Hofstede G. Dimensionalizing Cultures: The Hofstede Model in Context. Online Read Psychol Cult. 2011;2(1). 10.9707/2307-0919.1014

[13] Hofstede G. Culture’s consequences. London: Sage; 1980.

[14] Monea IS, Bengaa O, Opreb A. Cross-cultural differences in socialization goals as a function of power distance, individualism-collectivism and education. Rom J Exp Appl Psychol. 2016;7:330–4. doi: 10.15303/rjeap.2016.si1.a71

[15] Fiske ST. Controlling other people: The impact of power on stereotyping. Am Psychol. 1993;48:621–8. 10.1037/0003-066X.48.6.621 8328729 10.1037//0003-066x.48.6.621

[16] Heine SJ. Cultural psychology In Fiske ST, Gilbert DT, Lindzey G. editors. Handbook of Social Psychology. New York: John Wiley & Sons; 2010;1 p.1423–64.

[17] Hwang KK. Face and favor: The Chinese power game. Am J Sociol. 1987;92:944–74. 10.1086/228588

[18] Padilla-Walker LM, Carlo G, editors. Prosocial development: A multidimensional approach. New York, NY: Oxford University Press;2015.

[19] Bem DJ. Self-perception: An alternative interpretation of cognitive dissonance phenomena. Psychol Rev. 1967;74:183–200. 10.1037/h0024835 5342882 10.1037/h0024835

[20] Calderón-Tena CO, Knight GP, Carlo G. The socialization of prosocial behavioral tendencies among Mexican American adolescents: The role of familism values. Cultur Divers Ethnic Minor Psychol. 2011;17(1):98–106. 10.1037/a0021825 21341902 10.1037/a0021825

[21] Padilla-Walker LM, Fraser AM. How much is it going to cost me? Bidirectional relations between adolescents’ moral personality and prosocial behavior. J Adolesc. 2014;37:993–1001. 10.1016/j.adolescence.2014.07.008 25118040 10.1016/j.adolescence.2014.07.008

[22] Kasser T, Rosenblum KL, Sameroff AJ, Deci EL, Niemiec CP, Ryan RM, et al Changes in materialism, changes in psychological well-being: Evidence from three longitudinal studies and an intervention experiment. Motiv Emot. 2014;38(1):1–22. 10.1007/s11031-013-9371-4

[23] Yang Z, Fu X, Yu X, Lv Y. Longitudinal relations between adolescents’ materialism and prosocial behavior toward family, friends, and strangers. J Adolesc. 2018;62:162–70. 10.1016/j.adolescence.2017.11.013 29197702 10.1016/j.adolescence.2017.11.013

[24] Niranjan S, Gupta V, Goktan BA, Cheung YH, Gunay G, Pareek A. Cultural value orientation: Measurement invariance in a multi-country sample. J Manag Issues. 2013;25:264–83. 10.1037/t33366-000

[25] Little TD. Longitudinal structural equation modeling. New York, NY: Guilford press;2013.

[26] Peterson C, Seligman MEP. Character strengths and virtues: A handbook and classification. Washington, DC: Oxford University Press; 2004.

[27] Fu X, Kou Y, Yang Y. Materialistic values among Chinese adolescents: Effects of parental rejection and self-esteem. Child Youth Care Forum. 2015;44(1):43–57. 10.1007/s10566-014-9269-7

[28] Ajzen I. The theory of planned behavior. Organ Behav Hum Decis Process. 1991;56:179–211. 10.1016/0749-5978(91)90020-T

[29] Schwartz SH. Basic values: How they motivate and inhibit prosocial behavior In Mikulincer M, Shaver PR, editors. Prosocial motives, emotions, and behavior: The better angels of our nature. Washington, DC: American Psychological Association; 2010 p. 221–41.

[30] Carlo G, Padilla-Walker LM, Nielson MG. Longitudinal bidirectional relations between adolescents’ sympathy and prosocial behavior. Dev Psychol. 2015;51:1771–7. 10.1037/dev0000056 26414096 10.1037/dev0000056

[31] Fu X, Padilla-Walker LM., Brown MN. Longitudinal relations between adolescents’ self-esteem and prosocial behavior toward strangers, friends and family. J Adolesc. 2017;57:90–8. 10.1016/j.adolescence.2017.04.002 28402904 10.1016/j.adolescence.2017.04.002

[32] Hwang KK. Filial piety and loyalty: Two types of social identification in Confucianism. Asian J Soc Psychol. 1999;2(1):163–83. 10.1111/1467-839X.00031

[33] Murray C, Murray KM. Child level correlates of teacher–student relationships: An examination of demographic characteristics, academic orientations, and behavioral orientations. Psychol Sch. 2004;41(7):751–62. 10.1002/pits.20015

[34] Rudasill KM, Reio TG, Stipanovic N, Taylor JE. A longitudinal study of student–teacher relationship quality, difficult temperament, and risky behavior from childhood to early adolescence. J Sch Psychol. 2010;48(5):389–412. 10.1016/j.jsp.2010.05.001 20728689 10.1016/j.jsp.2010.05.001

[35] Löfdahl A, Hägglund S. Power and participation: Social representations among children in pre-school. Soc Psychol Educ. 2006;9(2):179–94. 10.1007/s11218-006-0002-8

[36] Brañas-Garza P, Capraro V, Ramírez ER. Gender differences in altruism on Mechanical Turk: Expectations and actual behaviour. Econ Lett. 2018;170:19–23. 10.1016/j.econlet.2018.05.022

[37] Rand DG, Brescoll VL, Everett JA, Capraro V, Barcelo H. Social heuristics and social roles: Intuition favors altruism for women but not for men. J Exp Psychol Gen. 2016;145(4):389–96. 10.1037/xge0000154 26913619 10.1037/xge0000154

[38] Rand DG. Social dilemma cooperation (unlike Dictator Game giving) is intuitive for men as well as women. J Exp Soc Psychol. 2017;73:164–8. 10.1016/j.jesp.2017.06.013 29686434 10.1016/j.jesp.2017.06.013 PMC5909828

[39] Capraro V. Gender differences in lying in sender-receiver games: A meta-analysis. Judgm Decis Mak. 2018;13(4):345–55. 10.2139/ssrn.2930944

